# Prevalence of metabolic syndrome in a cohort of Chinese schoolchildren: comparison of two definitions and assessment of adipokines as components by factor analysis

**DOI:** 10.1186/1471-2458-13-249

**Published:** 2013-03-21

**Authors:** Qiaoxuan Wang, Jinhua Yin, Lu Xu, Hong Cheng, Xiaoyuan Zhao, Hongding Xiang, Hugh Simon Lam, Jie Mi, Ming Li

**Affiliations:** 1 Department of Endocrinology, Key Laboratory of Endocrinology, Ministry of Health, Peking Union Medical College Hospital, Chinese Academy of Medical Sciences and Peking Union Medical College (CAMS & PUMC), Beijing, 100730, China; 2 Department of Epidemiology, Capital Institute of Pediatrics, Beijing, 100020, China; 3 Department of Paediatrics, Prince of Wales Hospital, The Chinese University of Hong Kong, Hong Kong, SAR, China

## Abstract

**Background:**

Although attention to metabolic syndrome (MetS) in children has increased, there is still no universally accepted definition and its pathogenesis remains unclear. Our aim was to compare the current definitions of childhood MetS in a Chinese cohort and to examine the clustering pattern of MetS risk factors, particularly inclusion of leptin and adiponectin as additional components.

**Methods:**

3373 schoolchildren aged 6 to 18 years were recruited. Anthropometric and biochemical parameters and adipokines were measured. MetS was identified using both the International Diabetes Federation (IDF) and a modified Adult Treatment Panel III (ATP III) definitions. Exploratory factor analysis was performed to establish grouping of metabolic characteristics.

**Results:**

For children ≥10 years, the prevalence of MetS was 14.3% in the obese group and 3.7% in the overweight group according to the new IDF definition, and 32.3% in the obese group and 8.4% in the overweight group according to the modified ATPIII definition. Frequency of hypertriglyceridemia, low high-density lipoprotein cholesterol (HDL-C), impaired fasting glucose, elevated blood pressure, and central obesity according to the new IDF definition was 16.7%, 20.7%, 15.8%, 25.5% and 75.5% in obese boys and 14.7%, 24.0%, 12.0%, 11.0% and 89.0% in obese girls, respectively. Metabolic abnormalities in children under 10 years of age were also noted. Using factor analysis on eight conventional variables led to the extraction of 3 factors. Waist circumference (WC) provided a connection between two factors in boys and all three factors in girls, suggesting its central role in the clustering of metabolic risk factors. Addition of leptin and adiponectin also led to the extraction of 3 factors, with leptin providing a connection between two factors in girls. When using WC, mean arterial pressure, triglyceride/HDL-C ratio, HOMA-IR and leptin/adiponectin ratio as variables, a single-factor model was extracted. WC had the biggest factor loading, followed by leptin/adiponectin ratio.

**Conclusions:**

MetS was highly prevalent amongst obese children and adolescents in this cohort, regardless of the definition used. Central obesity is the key player in the clustering of metabolic risk factors in children, supporting the new IDF definition. Moreover, our findings suggest that a common factor may underlie MetS. Leptin/adiponectin ratio as a possible component of MetS deserves further consideration.

## Background

Metabolic syndrome (MetS) refers to a combination of metabolic disturbances associated with type 2 diabetes mellitus (T2DM) and cardiovascular disease (CVD) [1]. In recent years, interest in childhood MetS has increased substantially due to the increasing prevalence of childhood obesity on a global scale. However, research in this area has been difficult in view of lack of a well-accepted definition of childhood MetS [2]. So far, most definitions have been adapted from the Adult Treatment Panel III (ATP III) definition [1], represented by the most commonly used version published by Cook et al. [2,3]. In order to estimate the global prevalence of MetS and make valid comparisons between nations, the International Diabetes Federation (IDF) released its definition of MetS in children and adolescents in 2007 (i.e., IDF 2007 definition) [4]. However, this definition has not yet been fully evaluated in different ethnic groups [5,6].

Recently, expansion of the definition to include other components such as adipokines and inflammatory marker levels has been considered [6]. It has been suggested that dysregulation of circulating adipokine levels may provide a link between obesity and insulin resistance, thereby influencing the development of MetS and vascular complications [7]. Leptin and adiponectin are the two major adipokines secreted by adipocytes, and have been recommended as adipose tissue biomarkers by IDF in their “platinum standard” definition of MetS for research [4]. Most studies have demonstrated that hyperleptinemia and hypoadiponectinemia are associated with the occurrence of T2DM, hypertension, dyslipidemia, MetS and CVD [7-14]. Moreover, some studies suggest that leptin/adiponectin ratio can be used as a potential atherogenic index [15] and provides adjunctive information for the risk of MetS [16]. However this has not been well studied in pediatric populations.

Although visceral obesity and insulin resistance appear to be at the core of the development of MetS, due to increasing numbers of metabolic risk factors and the complex interaction between the different components, the underlying pathogenesis of MetS remains unclear [2,4-6]. Factor analysis provides a means of condensing large numbers of highly intercorrelated variables into a few composite factors, thus allowing investigators to overcome analytic challenges, and help define the underlying structure [17]. Many studies [9-11,13,17-22] have used exploratory factor analysis to examine the associations between traditional and emerging risk factors considered to be potential components of MetS. However, with different components being used, so far no consistent conclusion has been reached. In recent years, it has been suggested that a one-factor model provided better understanding of MetS in studies using confirmatory factor analysis [23-25]. However, little is known about whether this one-factor model could be expanded to include other risk factors such as adipokine levels.

As there is so far no consensus over the definition of childhood MetS, we measured the prevalence of MetS in children and adolescents in Beijing, using both the new IDF definition and a modified ATPIII definition. Furthermore, based on data collected in the Beijing Child and Adolescent Metabolic Syndrome (BCAMS) study [26], we performed exploratory factor analysis on the various MetS components to characterize the clustering pattern and examine the role of leptin and adiponectin in the clustering of metabolic risk factors.

## Methods

### Subjects

Subjects were recruited from a cross-sectional population-based survey: the BCAMS cohort study [26,27]. A total of 3373 schoolchildren (1717 boys) were included in the current study, among them 637 were overweight and 1195 were obese. Age- and sex-specific body mass index (BMI) percentiles, developed by the Working Group for Obesity in China, were used to classify participants as normal weight (BMI <85%), overweight (BMI ≥85% but <95%), or obese (BMI ≥ 95%) [28]. Signed informed consent was obtained from participants and/or parents/guardians. The BCAMS study was approved by the Ethics Committee at the Capital Institute of Pediatrics in Beijing.

### Clinical and anthropometric measurements

Subjects’ height and weight were measured according to our standard protocol [28]. BMI was calculated as weight (kg) divided by height squared (m^2^). Waist circumference (WC) was measured midway between the lowest rib and the top of the iliac crest. The mean of 2 measurements made at the end of a normal expiration was used in the analyses. Two measurements of right arm systolic and diastolic blood pressure (SBP and DBP) were performed 3 times 10 minutes apart and the mean values of the latter 2 measurements were recorded. Pubertal development was assessed by Tanner stage of breast development in girls and testicular volume in boys [29]. Children with Tanner stage >1 were considered pubertal. This assessment was performed visually by two pediatricians of the same gender as the child.

### Laboratory measurement

Venous blood samples were collected after an overnight (≥12 h) fast. The samples were centrifuged, aliquoted and immediately frozen for future analysis of lipids and hormones. Blood samples were also analyzed for concentrations of plasma glucose, triglycerides (TG), total cholesterol (TC), high-density lipoprotein cholesterol (HDL-C), low-density lipoprotein cholesterol (LDL-C), insulin, adiponectin, and leptin. Serum lipids (enzymatic methods) and plasma glucose (glucose oxidase method) were assayed using the Hitachi 7060 C automatic biochemistry analysis system. HDL-C and LDL-C were measured directly. Insulin was measured by monoclonal antibody-based sandwich enzyme-linked immunosorbent assay (ELISA) [30], developed in the Key Laboratory of Endocrinology, Peking Union Medical College Hospital which had an inter-assay coefficients of variation (CV) of <9.0% and no cross-reactivity to proinsulin (<0.05%). Serum adiponectin was measured by ELISA with intra-assay and inter-assay CV of <5.4% and <8.5%, respectively; while serum leptin was measured by ELISA with intra-assay and inter-assay CV of <7.4% and <9.3%, respectively [26,31].

### Definitions

For this study, the presence of pediatric MetS was determined according to both the IDF 2007 definition [4] and a modified ATPIII definition [3,26] for comparison. The IDF 2007 definition was applied differently to different age groups: 6 to <10, 10 to <16, and ≥16 years. For children aged 10 to <16 years, a diagnosis of MetS was made as the presence of abdominal obesity (WC ≥ 90th percentile for age and gender or adult cut-off if lower) plus the presence of two or more of the other components: elevated TG (≥1.7 mmol/L), low HDL-C (<1.03 mmol/L), high blood pressure (systolic ≥130 mmHg or diastolic ≥85 mmHg), and elevated blood glucose (≥ 5.6 mmol/L). IDF criteria for adults [4] were used to identify MetS in those aged ≥16 years. Although the IDF definition was not intended to be applied to children below 10 years of age, for the purposes of this study to enable comparisons to be made, we have defined the individual risk components of MetS as for children aged between 10 to <16 years [4,32].

For comparison, a modified ATPIII definition was also employed in which MetS was defined by the presence of three or more of the following five components [3,26]: (1) central obesity defined as WC ≥ 90th percentile for age and gender (established based on the BCAMS study); (2) elevated systolic and/or diastolic blood pressure ≥ 90th percentile for age, sex and height (according to the BCAMS study); (3) hypertriglyceridemia defined as TG ≥1.24 mmol/L, equal to the 90th percentile of the reference population; (4) low serum HDL-C (Low-HDL) defined as ≤1.03 mmol/L i.e., ≤ 5th percentile of the reference population and (5) impaired fasting glucose (IFG) defined as ≥ 5.6 mmol/L.

Insulin resistance index was calculated by homeostasis model assessment of insulin resistance (HOMA-IR) as (fasting insulin IU/L) × (fasting glucose mmol/L)/22.5. Insulin resistance was defined as HOMA-IR ≥3.0, i.e., ≥95th percentile of the reference population (subjects without any of the “conventional” MetS components from the BCAMS study). Hyperleptinemia was defined as leptin ≥ 95th percentile and hypoadiponectinemia was defined as adiponectin ≤ 5th percentile for gender of the same reference population.

### Statistical analysis

Data were analyzed using SPSS for Windows 15.0 (SPSS Inc., Chicago, IL, USA). All skewed distributions were log transformed for analysis. Results are expressed as mean ± standard deviation (SD) or percentage (%) as appropriate. The mean values of variables studied were compared between normal weight, overweight and obese groups by analysis of variance (ANOVA) and post hoc analysis. Prevalence of individual metabolic abnormalities of different groups was compared using the χ2 test or Fisher’s exact test as appropriate. Correlations were calculated using Pearson correlation coefficient.

According to previous studies [17,33,34], exploratory factor analysis is well suited for determining the clustering patterns of MetS components and helping suggest underlying pathophysiology. Exploratory factor analysis was performed using principal component analysis, a technique for reducing the number of original variables into fewer latent factors. The factors are created by a scoring algorithm such that individual variables “load” most strongly onto the factor with which they are most correlated. The factor loading of a variable on a factor equals the Pearson correlation coefficient between that variable and the factor. Thus, higher factor loadings represent more correlation between the variable and the latent factor. In MetS, if the analysis reveals only one underlying factor, this may be interpreted as supporting the theory that one unifying physiology accounts for the metabolic risk variables clustering, thus favoring a common pathogenesis. The unity hypothesis would be less probable if more than one factor is found. However, if a measured risk variable is associated with more than one factor, this overlap reveals unifying commonalties between physiologic domains. Thus, the patterns of overlap could provide valuable insights into the underlying structure of the syndrome [17,22,33].

Factor analysis includes three steps : (1) factor extraction, which produces the minimum number of factors that retain as much of the total variance in the original data as possible; (2) varimax rotation to make factors more readily interpretable; and (3) interpretation based on rotated factor loadings. In this study, factors with an eigenvalue (the amount of variance attributable to the factor) of greater than one were extracted. Factor loadings of ≥ |0.40| were considered meaningful for interpretation, because this ensures that the variable shares at least 15% of the variance with the factor [17.25]. The analysis was performed first on the conventional metabolic variables (WC, DBP, SBP, HOMA-IR, insulin, glucose, TG and HDL-C) and subsequently with the addition of leptin and adiponectin. Since factor analysis extracts factors due to the interrelatedness of measured variables, using two or more measures for the same trait (e.g. SBP and DBP, TG and HDL-C) would increase the number of factors found [24,33]. We then used one parameter for each of the postulated MetS components: WC for central obesity and mean arterial pressure (MAP) for blood pressure, TG/HDL-C ratio for dyslipidemia trait, and HOMA-IR for insulin resistance. Using these three composite parameters along with WC, a third factor analysis was performed. After that, leptin/adiponectin ratio was employed as an additional component for the fourth factor analysis. Bartlett’s test of sphericity was used to examine the appropriateness of using factor analysis. In this study, p values for Bartlett’s test of sphericity were less than 0.001.

## Results

### Descriptive statistics

Anthropometric and metabolic parameters of the study population by gender and BMI classification are presented in Table 1. This study included 3373 children and adolescents (1717 boys, 1656 girls) aged 6–18 years. The mean ages ± SD of boys and girls were 11.8 ± 3.0 and 12.2 ± 3.0 years, respectively (P < 0.001). More boys were obese than girls (P < 0.001). Compared to the normal weight group, mean WC, SBP, DBP, TG, insulin and HOMA-IR were significantly higher, while HDL-C was significantly lower in obese children. As BMI increased, so did leptin concentrations and leptin/adiponectin ratios.

**Table 1:** Characteristics of the study population according to BMI category in boys and girls

	**Boys**			**Girls**		
	**Normal weight**	**Overweight**	**Obese**	**Normal weight**	**Overweight**	**Obese**
N	629	319	769	912	318	426
Age (ys)	11.7 ± 3.1^a^	12.7 ± 3.1^b^	11.4 ± 2.9^a^	12.3 ± 3.1^a^	12.9 ± 2.9^b^	11.2 ± 2.9^c^
Tanner stage	2.1 ± 1.2^a^	2.6 ± 1.3^b^	2.1 ± 1.2^a^	3.1 ± 1.3^a^	3.8 ± 1.4^b^	3.1 ± 1.5^a^
BMI (kg m^-2^)	17.7 ± 2.4^a^	23.3 ± 2.7^b^	26.9 ± 3.6^c^	18.0 ± 2.4^a^	23.4 ± 2.3^b^	25.9 ± 3.8^c^
WC (cm)	63.3 ± 7.6^a^	78.2 ± 8.8^b^	86.1 ± 10.8^c^	62.1 ± 6.6^a^	74.0 ± 6.3^b^	79.5 ± 9.7^c^
SBP (mm Hg)	103 ± 13^a^	114 ± 14^b^	116 ± 13^c^	101 ± 12^a^	109 ± 10^b^	111 ± 11^c^
DBP (mm Hg)	64 ± 10^a^	70 ± 10^b^	72 ± 9^c^	64 ± 9^a^	69 ± 8^b^	71 ± 9^c^
TC (mmol/L)	4.03 ± 0.84	4.03 ± 0.76	4.11 ± 0.75	4.18 ± 0.86^a^	4.05 ± 0.77^b^	4.04 ± 0.73^b^
TG (mmol/L)^#^	0.79 ± 0.39^a^	1.08 ± 0.58^b^	1.20 ± 0.63^c^	0.96 ± 0.50^a^	1.05 ± 0.54^b^	1.18 ± 0.63^c^
HDL-C (mmol/L)	1.55 ± 0.36^a^	1.33 ± 0.32^b^	1.27 ± 0.26^c^	1.52 ± 0.30^a^	1.34 ± 0.26^b^	1.26 ± 0.26^c^
LDL-C (mmol/L)	2.40 ± 0.76^a^	2.52 ± 0.67^a^	2.65 ± 0.66^b^	2.56 ± 0.80	2.56 ± 0.70	2.58 ± 0.66
Glucose (mmol/L)	5.12 ± 0.66	5.17 ± 0.47	5.19 ± 0.48	4.96 ± 0.44^a^	5.15 ± 1.02^b^	5.11 ± 0.50^b^
Insulin (mU/L) ^#^	6.2 ± 4.3^a^	10.6 ± 7.7^b^	14.8 ± 13.9^c^	7.3 ± 4.9^a^	11.6 ± 6.9^b^	15.4 ± 12.5^c^
HOMA-IR^#^	1.42 ± 1.01^a^	2.50 ± 2.09^b^	3.52 ± 3.80^c^	1.65 ± 1.20^a^	2.67 ± 1.62^b^	3.57 ± 3.34^c^
Leptin (ng/ml)^#^	1.7 ± 2.5^a^	7.9 ± 9.1^b^	15.6 ± 12.3^c^	5.3 ± 5.8^a^	14.6 ± 10.8^b^	20.5 ± 14.0^c^
Adiponectin (μg/L)^#^	15.1 ± 8.7^a^	11.4 ± 7.3^b^	10.3 ± 5.8^b^	15.0 ± 7.8^a^	11.5 ± 5.8^b^	10.6 ± 5.3^b^
Leptin/Adiponectin^#^	0.18 ± 0.39^a^	0.95 ± 1.24^b^	2.04 ± 1.99^c^	0.55 ± 0.95^a^	1.74 ± 2.21^b^	2.63 ± 2.83^c^

Abbreviations: *BMI* body mass index, *WC* waist circumference, *SBP* systolic blood pressure, *DBP* diastolic blood pressure, *TC* total cholesterol, *TG* triglycerides, *HDL-C* high-density lipoprotein cholesterol, *LDL-C* low-density lipoprotein cholesterol, *HOMA-IR* homeostatic model assessment of insulin resistance, *leptin/adiponectin* leptin-to-adiponectin ratio. Data are expressed as mean ± SD. ^#^Skewed distributions were logarithmically transformed for one-way ANOVA. Values in the same row with different superscript letters (e.g. a, b, c) are significantly different, with p < 0.05 (one-way ANOVA between normal-weight vs. overweight vs. obese groups).

### Prevalence of MetS and individual MetS abnormalities

As the IDF definition suggests that MetS should not be diagnosed below the age of 10 years (4), we have divided our population into two groups for analysis: ≥ 10 years, and <10 years. Gender-specific prevalence of individual MetS abnormalities by BMI category according to both the IDF 2007 and modified ATPIII definitions was estimated (Table 2 and Table 3).

**Table 2:** Prevalence of individual metabolic components in children ≥ 10 years according to BMI category

	**IDF 2007 definition**				**Modified ATPIII definition**			
	**Normal weight**	**Overweight**	**Obese**	**P**	**Normal weight**	**Overweight**	**Obese**	**P**
Boys (n)	464	269	546		464	269	546	
Hyper-TG	13(2.8%)	34(12.6%)	91(16.7%)	<0.001	43(9.3%)	82(30.5%)	205(37.5%)	<0.001
Low-HDL	24(5.2%)	40(14.9%)	113(20.7%)	<0.001	26(5.6%)	42(15.6%)	120(22.0%)	<0.001
IFG	74(15.9%)	39(14.5%)	86(15.8%)	NS	74(15.9%)	39(14.5%)	86(15.8%)	NS
Elevated BP	22(4.7%)	36(13.4%)	139(25.5%)	<0.001	32(6.9%)	54(20.1%)	202(37.0%)	<0.001
Central obesity	0	30(11.2%)	412(75.5%)	<0.001	0	41(15.2%)	416(76.2%)	<0.001
MetS (%)	0	7(2.6%)	80(14.7%)	<0.001	2(0.4%)	22(8.2%)	184(33.7%)	<0.001
Girls (n)	725	268	292		725	268	292	
Hyper-TG	54(7.4%)	29(10.8%)	43(14.7%)	0.002	151(20.8%)	66(24.6%)	102(34.9%)	<0.001
Low-HDL	63(8.7%)	52(19.4%)	70(24.0%)	<0.001	32(4.4%)	31(11.6%)	55(18.8%)	<0.001
IFG	56(7.7%)	33(12.3%)	35(12.0%)	0.029	56(7.7%)	33(12.3%)	35(12.0%)	0.029
Elevated BP	16(2.2%)	10(3.7%)	32(11.0%)	<0.001	61(8.4%)	44(16.4%)	104(35.6%)	<0.001
Central obesity	8(1.1%)	96(35.8%)	260(89.0%)	<0.001	8(1.1%)	110(41.0%)	260(89.0%)	<0.001
MetS (%)	0	13(4.9%)	40(13.7%)	<0.001	10(1.4%)	24(9.0%)	89(30.5%)	<0.001

Abbreviations: *IDF* International Diabetes Federation, *ATPIII* Adult Treatment Panel III, *Hyper-TG* Hypertriglyceridemia, *Low-HDL* low serum HDL cholesterol, *IFG* impaired fasting glucose, *BP* Blood pressure, *MetS* Metabolic syndrome.

Prevalence of individual metabolic component was estimated using the IDF 2007 and the modified ATPIII definitions. The IDF definition for children from 10 to 16 years included the presence of central obesity (WC ≥ 90th percentile) plus two of the other four factors: (1) Hyper-TG with fasting TG ≥1.7 mmol/L, (2) Low-HDL with HDL-C <1.03 mmol/L, (3) hypertension with SBP ≥ 130 and/or DBP ≥ 85 mm Hg, (4) IFG with fasting glucose ≥ 5.6 mmol/L. IDF criteria for adults were used to define MetS in those aged ≥16 years. The modified ATPIII definition for children was defined by the presence of three or more of the following five components: (1) central obesity (WC ≥ 90th percentile), (2) elevated SBP and/or DBP ≥ 90th percentile, (3) fasting TG ≥ 1.24 mmol/L, (4) HDL-C ≤ 1.03 mmol/L, and (5) fasting glucose ≥ 5.6 mmol/L.

Results are expressed as number with percentage (%). Significance was calculated by χ2 test or Fisher’s exact test among normal weight, over weight and obese groups in both genders.

**Table 3:** Prevalence of individual metabolic components in children<10 years according to BMI category

	**IDF 2007 definition**				**Modified ATPIII definition**			
	**Normal weight**	**Over-weight**	**Obese**	**P**	**Normal weight**	**Over-weight**	**Obese**	**P**
Boys (n)	165	50	223		165	50	223	
Hyper-TG	7(4.2%)	5(10.0%)	34(15.2%)	0.002	16(9.7%)	10(20.0%)	73(32.7%)	<0.001
Low-HDL	2(1.2%)	2(4.0%)	14(6.3%)	0.043	2(1.2%)	2 (4.0%)	14(6.3%)	0.034
IFG	18(10.9%)	9(18.0%)	33(14.8%)	NS	18(10.9%)	9 (18.0%)	33(14.8%)	NS
Elevated BP	4(2.4%)	1(2.0%)	15(6.7%)	NS	14(8.5%)	6(12.0%)	60(26.9%)	<0.001
Central obesity	4(2.4%)	5(10.0%)	181(81.2%)	<0.001	4(2.4%)	5(10.0%)	181(81.2%)	<0.001
MetS (%)	0	0	14(6.3%)	<0.001	2(1.2%)	3(6.0%)	42(18.8%)	<0.001
Girls (n)	187	50	134		187	50	134	
Hyper-TG	6(3.2%)	1(2.0%)	13(9.7%)	0.032	23(12.3%)	8(16.0%)	43(32.1%)	<0.001
Low-HDL	6(3.2%)	4(8.0%)	16(11.9%)	0.008	7(3.7%)	4 (8.0%)	18(13.4%)	0.005
IFG	3(1.6%)	5(10.0%)	10(7.5%)	0.005	3(1.6%)	5 (10.0%)	10(7.5%)	0.005
Elevated BP	5(2.7%)	3(6.0%)	6(4.5%)	NS	17(9.1%)	11 (22.0%)	38(28.4%)	<0.001
Central obesity	8(4.3%)	21(42.0%)	114(85.1%)	<0.001	8(4.3%)	21 (42.0%)	114(85.1%)	<0.001
MetS (%)	0	0	8(6.0%)	<0.001	2(1.1%)	0	36(26.9%)	<0.001

Abbreviations: *IDF* International Diabetes Federation, *ATPIII* Adult Treatment Panel III, *Hyper-TG* Hypertriglyceridemia, *Low-HDL* low serum HDL cholesterol, *IFG* impaired fasting glucose, *BP* Blood pressure, *MetS* metabolic syndrome.

Prevalence of individual metabolic component in these children aged below 10 years was estimated using the IDF definition for children aged 10 to 16 years and the modified ATPIII definitions, respectively. The IDF definition for children from 10 to 16 years included the presence of central obesity (WC ≥ 90th percentile) plus two of the other four factors: (1) Hyper-TG with fasting TG ≥1.7 mmol/L, (2) Low-HDL with HDL-C <1.03 mmol/L, (3) hypertension with SBP ≥ 130 and/or DBP ≥ 85 mm Hg, (4) IFG with fasting glucose ≥ 5.6 mmol/L. The modified ATPIII definition for children was defined by the presence of three or more of the following five components: (1) central obesity (WC ≥ 90th percentile), (2) elevated SBP and/or DBP ≥ 90th percentile, (3) fasting TG ≥ 1.24 mmol/L, (4) HDL-C ≤ 1.03 mmol/L, and (5) fasting glucose ≥ 5.6 mmol/L. Results are expressed as number with percentage (%). Significance was calculated by χ2 test or Fisher’s exact test among normal weight, over weight and obese groups in both genders.

For girls and boys older than 10 years, prevalence of MetS was 14.3% in the obese group and 3.7% in the overweight group according to the IDF 2007 definition. Central obesity was the most common abnormality in obese children in both genders, followed by elevated blood pressure in boys and Low-HDL in girls. According to the modified ATPIII definition, prevalence of MetS was 32.3% in obese children and 8.4% in the overweight, respectively. The most common component of MetS was still central obesity, followed by hypertriglyceridemia and elevated blood pressure in boys and girls, respectively. Apart from the normal weight group of boys, prevalence of MetS differed significantly depending on which of the two definitions was used for diagnosis. Prevalence of other metabolic abnormalities including insulin resistance, hyperleptinemia and hypoadipotinemia, also increased significantly with increase in body weight, with the highest percentages in the obese group (52.5%, 81.2% and 16% respectively) (see Additional file 1).

For children < 10 years of ages, according to the ATPIII definition, the prevalence of MetS in obese group was still high (18.8% in boys and 26.9% in girls) (Table 3). To provide a comparison, we also used the IDF definition for children aged 10 to 16 years to estimate the metabolic abnormalities; the prevalence of MetS in the obese group dropped to 6.2% (Table 3). In addition, prevalence of hyperleptinemia was 83.4% and 46.3% in obese boys and girls, respectively; while prevalence of insulin resistance was 20.2% and 22.4%, respectively, which was much lower than in older children (see Additional file 1).

### Analysis of correlation

The correlation matrix between the variables of interest is shown in Additional file 1. Serum leptin level was significantly associated with all conventional variables of MetS, especially WC and insulin in both genders. Serum adiponectin level had positive correlation with HDL-C and negative correlation with WC, TG, SBP and DBP in boys as well as girls. WC had the strongest association with leptin/adiponectin and was strongly associated with leptin, insulin, HOMA-IR, HDL-C, SBP and DBP in both genders. Apart from insulin, WC and leptin had the strongest positive correlation with HOMA-IR in boys and girls respectively. Considering lipid parameters in both genders, HDL-C had the strongest positive and negative correlation with adiponectin and WC, respectively, while TG had the strongest positive correlation with leptin/adiponectin.

### Exploratory factor analysis

Table 4 provides the results of the factor analysis with and without leptin and adiponectin as variables in both genders. Entering the conventional components of MetS (WC, SBP, DBP, TG, HDL-C, glucose, HOMA-IR and insulin), exploratory factor analysis extracted three factors with some differences in both genders (accounting for 76.8% and 73.0% of the total variance in boys and girls, respectively). In boys, WC, TG, HOMA-IR and insulin contributed positively on factor 1, and HDL-C contributed negatively on this factor. WC, SBP and DBP had positive contributions on factor 2. HOMA-IR, insulin and glucose showed positive contributions to factor 3. In girls, TG, HDL-C, HOMA-IR and insulin were not loaded together, but on factor 1 and 3 separately. WC together with HOMA-IR, insulin and glucose constituted the first factor which accounted for the largest proportion of the total variance in girls. Similar to boys, factor 2 was mainly determined by SBP, DBP and WC. Notably, WC was loaded on two of the three factors in boys and all three factors in girls, while HOMA-IR and insulin loaded into two factors in boys but only one factor in girls. This finding may indicate that WC has a unifying role, more so than HOMA-IR or insulin in the clustering of MetS, in girls.

**Table 4:** Factor loadings of conventional variables with leptin and adiponectin on the selected factors

**Variable**	**Conventional variables**			**Conventional variables**		
				**+leptin + adiponectin**		
	**Factor 1**	**Factor 2**	**Factor 3**	**Factor 1**	**Factor 2**	**Factor 3**
Boys (n = 1717)						
WC	**0.616**	**0.557**	0.158	**0.701**	**0.495**	0.136
SBP	0.246	**0.876**	0.143	0.287	**0.864**	0.140
DBP	0.110	**0.906**	0.087	0.143	**0.898**	0.110
TG#	**0.753**	0.095	0.037	**0.696**	0.060	0.072
HDL-C	**−0.752**	−0.223	0.166	**−0.695**	−0.213	0.184
Glucose	−0.149	0.092	**0.831**	−0.127	0.118	**0.819**
HOMA-IR^#^	**0.648**	0.210	**0.676**	**0.670**	0.171	**0.650**
Insulin^#^	**0.689**	0.207	**0.600**	**0.710**	0.163	**0.574**
Leptin^#^				**0.707**	0.160	0.239
Adiponectin^#^				**−0.546**	−0.237	0.159
Variance explained (%)	31.275	25.651	19.874	33.284	19.987	15.937
Cumulative variance (%)	31.275	56.926	76.800	33.284	53.271	69.208
Girls (n = 1656)						
WC	**0.419**	**0.510**	**0.447**	**0.425**	**0.547**	**0.469**
SBP	0.167	**0.891**	0.093	0.173	0.139	**0.877**
DBP	0.127	**0.891**	0.031	0.147	0.059	**0.883**
TG^#^	0.180	−0.057	**0.763**	0.248	**0.625**	−0.130
HDL-C	0.017	−0.205	**−0.805**	0.002	**−0.759**	−0.127
Glucose	**0.681**	0.018	−0.131	**0.670**	−0.169	0.035
HOMA-IR^#^	**0.859**	0.280	0.321	**0.852**	0.340	0.246
Insulin^#^	**0.803**	0.288	0.356	**0.797**	0.381	0.250
Leptin^#^				**0.491**	**0.529**	0.350
Adiopnectin^#^				−0.008	**−0.614**	−0.173
Variance explained (%)	26.228	25.699	21.097	23.449	22.352	20.796
Cumulative variance (%)	26.228	51.927	73.024	23.449	45.801	66.597

Abbreviations: *WC* waist circumference, *SBP* systolic blood pressure, *DBP* diastolic blood pressure, *TG* triglycerides, *HDL-C* high-density lipoprotein cholesterol, *HOMA-IR* homeostatic model assessment of insulin resistance. ^#^Skewed distributions were logarithmically transformed. Factors with an eigenvalue ≥1 were selected for analysis. Factor loading ≥ |0.40| are in bold font.

**Figure 1:**
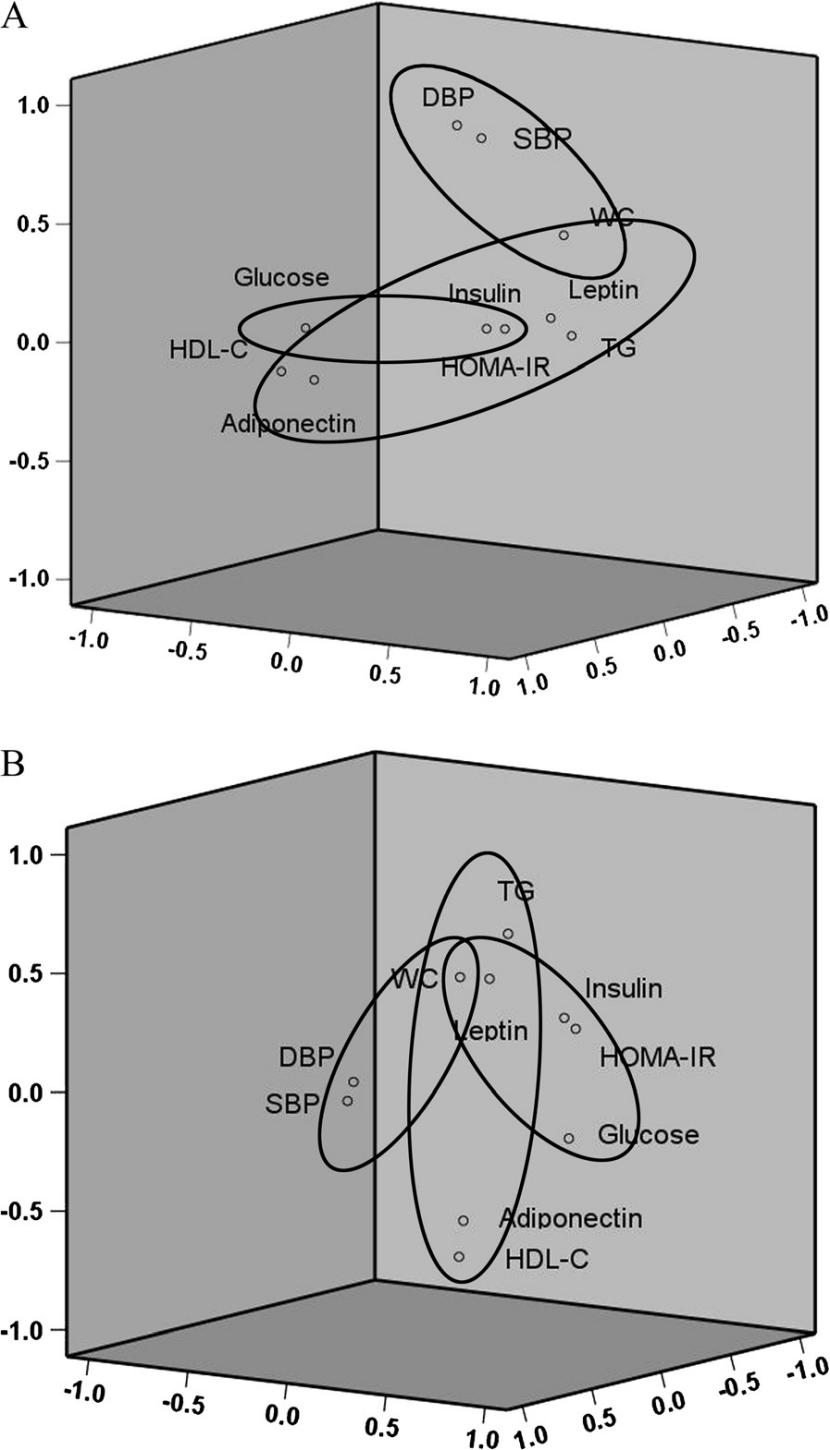
**Component plots with factor diagrams from exploratory factor analysis with varimax rotation.** Classic variables, leptin and adiponectin were all included. **A**, boys (n = 1717). **B**, girl (n = 1656).

The factor structures showed some differences when analyzed together with leptin and adiponectin in girls but not in boys (Figure 1). Again, three factor structures were retained which explained 69.2% and 66.6% of the total variance in boys and girls, respectively. In boys, leptin loaded positively on the first factor associated with WC, TG, HDL-C, HOMA-IR and insulin, while adiponectin loaded negatively on this factor. WC and HOMA-IR still retained their loadings on two factors. In girls, HOMA-IR, insulin and glucose still loaded on the first factor; leptin showed positive loading in both factor 1 and 2, while adiponectin showed negative loading only in the second factor together with TG and HDL-C; the unifying role of WC was still retained, and as it was for leptin if relaxation of the factor loading threshold to 0.30 was allowed.

Results of factor analysis with only four (WC, MAP, TG/HDL-C, HOMA-IR) or five parameters (adding leptin/adiponectin ratio as additional variable) are presented in Table 5. One-factor model which was extracted in both genders with or without leptin/adiponectin, explained 61.7% and 55.8%-58% of the total variance in boys and girls, respectively. In these two models, WC had the biggest factor loading, while in the one with leptin/adiponectin, this parameter made the second largest contribution. In addition, after further stratifying for pubertal stage (prepubertal and pubertal), the one-factor model was still consistent and explained a similar amount of variance in both genders (data not shown).

**Table 5:** Factor loadings of conventional variables with leptin/adiponectin on the selected factors among boys and girls

	**Boys (n = 1717)**		**Girls (n = 1656)**	
	**Conventional variables**	**Conventional variables + Leptin/Adiponectin**	**Conventional variables**	**Conventional variables + Leptin/Adiponectin**
Variable	Factor 1	Factor 1	Factor 1	Factor 1
WC	0.873	0.890	0.851	0.875
MAP	0.737	0.675	0.693	0.635
TG/HDL-C^#^	0.792	0.786	0.820	0.804
Leptin/Adiponectin^#^	-	0.842	-	0.869
Variance explained (%)	61.7	61.7	55.8	58.0

Abbreviations: *WC* waist circumference, *MAP* mean arterial pressure, *TG* triglycerides, *HDL-C* high-density lipoprotein cholesterol, *TG/HDL* TG-to-HDL ratio, *HOMA-IR* homeostatic model assessment of insulin resistance, *Leptin/Adiponectin* leptin-to-adiponectin ratio. ^#^ Skewed distributions were logarithmically transformed. Factors with an eigenvalue ≥1 were selected for analysis.

## Discussion

### Comparison between these two definitions

Because there is still no universally accepted definition of childhood MetS, pediatric studies have invariably adapted adult standards in conjunction with use of gender- and age-dependent normal values(2). In this study, we applied both the IDF and modified ATPIII definitions to the same population to estimate prevalence of MetS in a sample of overweight and obese children in Beijing.

Our results show that prevalence of MetS using the IDF 2007 definition is lower in obese children in Beijing (14.3%) compared to their counterparts in the USA (20.8%), Korea (24.3%) [35] and Turkey (31%) [36], but higher than children in Greece (7.7%) [37]. However, these estimates should be interpreted with caution since the various studies were not standardized with respect to ethnicity, pubertal status, age range, sample size and collection methods.

For children ≥10 years of age in our study, the prevalence estimated according to the IDF 2007 definition was significantly lower than the modified ATPIII definition estimate. It is obvious that the sensitivity of the IDF definition for diagnosing of MetS is significantly lower than the modified ATPIII definition, especially in the overweight and obese groups, because these two definitions although included the same components, had different cut-points for hypertriglyceridemia and elevated blood pressure. In addition, the IDF definition positions central obesity as an essential element, which almost rules out the possibility of diagnosing MetS in a normal weight individual.

Another important discrepancy of these definitions is that the IDF definition suggests the diagnosis of MetS not be made for children below the age of 10 years [4]. In these children we applied the modified ATPIII criteria to define MetS and still found a high prevalence among obese children (21.8%). In addition, as have been performed in other studies [32], we applied the IDF criteria for children aged 10 to 16 years to this younger group. Though this may lead to underestimation, we still found about 6% of obese children meeting the criteria for MetS. These results show that early-onset MetS is of concern. Regardless of the definition of MetS, there is increasing prevalence of MetS features in this young age group with increasing obesity, with potential long-term adverse consequences.

It has been reported that diagnosis of childhood MetS lacked stability. In two previous studies, approximately half of the adolescents with MetS at baseline no longer had the diagnosis at follow-up [38,39]. This could be due to the lack of consistency in the pediatric definition and/or the instability of the clinical features in children which are used to define MetS in adults. Currently, apart from the IDF, most pediatric definitions define their components such as the abnormalities of lipids, BPs on the base of the age-, sex-specific percentiles rather than absolute numbers [4,5]. This may result in fluctuation in the individual diagnosis of MetS as the child’s clinical parameters develop at a different rate than the “normal” population. In addition, without long-term outcomes data, by simply basing the diagnosis of MetS on predominantly percentile-based criteria will cause problems as the overall population’s clinical parameters worsen, or improve with time.

### Factor structures of MetS

Exploratory factor analysis resolves highly interrelated variables into a set of unrelated latent factors and is particularly useful for examining the nature of MetS. In this study, factor analysis conducted with conventional components of MetS led to extraction of three factors in both genders, which was consistent with previous pediatric studies [20,21]. Previous factor analysis performed among 934 non-diabetic and 305 diabetic Chinese adults led to extraction of four factors [18]. The factor structures seen in our study were similar to those seen in adults, suggesting that similar unfavorable interactions between risk factors of cardiovascular disease and T2DM are established early in life [40].

Models with more than one factor are suggestive of complex etiology of MetS and may appear to contradict the unity hypothesis [17]. However, a previous study has pointed out that using two or more measures for the same trait would ensure that highly correlated measures cluster together under a separate factor rather than load on a common factor [23], and studies with a higher starting number of components tended to produce a larger number of factors [2,23]. These effects were confirmed in this study. With only one measure for each postulated MetS component, a one-factor model was also obtained in our study, suggesting that there may indeed be a common causal factor that underlies these different components of MetS [24,25,27]. Considering that parameters loading on the same factor are strongly associated with each other [34], this one-factor model is therefore not necessarily a contradiction of a three-factor model that has a larger number of observed variables. With leptin/adiponectin ratio as an additional component, the one-factor model was still retained. Previous studies with one-factor models [24,25,27] differed from ours in that they all used confirmatory factor analysis and none of them included leptin/adiponectin as a variable.

### Associations between adipokines and MetS

Leptin and adiponectin are two well-studied adipokines [7]. However, whether the two adipokines should be considered as additional MetS components is controversial. Several studies have conducted factor analysis to provide support [9-11]. In our study, hyperleptinemia (perhaps leptin resistance) is common amongst obese children. Further, leptin level was associated with insulin resistance and all other components of MetS. In factor analysis, if the factor loading threshold used for interpretation was reduced from 0.4 to 0.3 [17,33], leptin would have loaded onto all three factors in girls, thus, potentially playing a unifying role. This result is in contrast to previous studies [9-11]. In a study conducted in Canadian children, leptin loaded only onto an adiposity factor [41]. In a large adult study conducted in Mauritius, leptin loaded onto a major factor containing BMI, WC, insulin, TG and HDL-C [11]. To our knowledge, no unifying role of leptin has been reported. However, previous studies have shown that high plasma leptin level is associated with the development of essential hypertension, hyperinsulinemia and dyslipidemia [7,42]. Therefore it should not be surprising that leptin could align with the lipid, glucose and blood pressure factors. Furthermore, previous studies were often carried out in community-based or school-based populations, in which leptin resistance induced by hyperleptinemia was not commonly present and the effect of both hyperleptinemia and leptin resistance on MetS was not yet apparent. In contrast, with a large sample of children at risk for MetS from the BCAMS study, prevalence of overweight and obesity was much higher than in the general population. This made it easier to reveal the adverse associations of hyperleptinemia. As indicated in our study, girls were found to have significantly higher leptin concentrations than boys at the same degree of adiposity [43], and this may partly explain why the unifying role of leptin was more obvious in girls compared to boys. In addition, when analysis was stratified by pubertal status, the role of leptin on linking the conventional components of MetS became more apparent in pubertal than in prepubertal children, regardless of their gender (data not shown).

Although secreted predominantly by adipose tissue, concentration of adiponectin, unlike that of other adipokines, is decreased in obesity [6,7]. Adiponectin has properties that have been characterized as antiatherogenic, antidiabetic, and anti-inflammatory (7). Hypoadiponectinemia has also shown to be associated with the occurrence of MetS, T2DM, hypertension, dyslipidemia and CVD [6,7,12,42]. Although factor structures obtained in previous studies [13,41] carried out on children and adults showed some differences, as with our results, clustering of adiponectin together with TG and HDL-C is commonly observed. As with other studies [13,41,44], we found that adiponectin was significantly related to SBP and DBP in correlation analysis, but did not load onto the same factor with these variables. However, unlike leptin, adiponectin loaded only on one factor suggesting that it may not play a causative role in the clustering of metabolic abnormalities in children.

Recently, leptin/adiponectin was proposed as a biomarker that may serve as a potential atherogenic index in obese T2DM patients [15], and it provides adjunctive information to the risk of MS beyond the HOMA-IR [16]. In our one-factor model, leptin/adiponectin was, after WC, the second highest factor loading, followed by HOMA-IR, MAP or TG/HDL-C. Moreover, this one-factor model was not altered after stratification by pubertal status, and thus appeared to be consistent across sex and puberty. This finding is consistent with another recent report, which also revealed the stability of the one-factor structure of MetS from childhood to adolescence during a 6-year follow-up study [45]. Therefore, pubertal development may have impact on adipokine secretion [46], but may not affect the stability of a single-factor model of MetS with leptin/adiponectin as a variable.

Our factor analysis results suggest that leptin/adiponectin should be considered a component of MetS. However, further follow up studies of leptin, adiponectin and their ratio in children and adolescents are needed to determine if they could (i) be predictors of MetS in adulthood, (ii) increase the stability of MetS diagnosis in children and (iii) provide additive value to predicting future cardiovascular morbidity.

### Associations between WC and MetS

Apart from glucose, WC was associated with all MetS risk factors, and its correlation with most of these variables was much stronger than HOMA-IR. With or without adipokines as variables, WC loaded onto the first two factors in boys and all three factors in girls, while HOMA-IR loaded onto only two factors in boys and one in girls. In the one-factor model, WC contributed most (with highest loading factor), while HOMA-IR contributed less. HOMA-IR contributed less than leptin/adiponectin ratio. These results implicate that central obesity in children plays a more important role in unifying risk factors for MetS than insulin resistance, and that insulin resistance may be causally downstream of central obesity [47], supporting the IDF 2007 definition that central obesity should be taken as the essential element in MetS. Our results are compatible with the theory that MetS is related primarily to dysregulation of adipose tissue [47]. Visceral fat accumulation results in adipose tissue dysfunction, which in turn leads to over secretion of deleterious adipokines and hyposecretion of beneficial adipokines such as adiponectin. This may be one of the major underlying mechanisms in obesity-related diseases [7], explaining the clustering of WC together with hyperleptinemia and hypoadiponectin.

Identifying high risk children is important to help prevent them from future development of MetS. As an anthropometric measurement which is easy to perform, there is no doubt that WC would be a good tool for screening children at risk of MetS [4]. However, some concerns remain. First, it should be noted that even normal-weight individuals may develop features of MetS. This makes it difficult to identify the appropriate cut-points for WC above which it would be considered a risk factor. Second, although abdominal obesity can be easily assessed using the simple measure of WC, other complex measures such as the body fat distribution (by dual energy X-ray absorptiometry or magnetic resonance imaging), or adipose tissue biomarkers such as leptin and adiponectin, may be appropriate surrogate markers of central obesity and should play an important role in childhood MetS research [4]. Notably, since unfavorable changes in secretion of adipokines, such as elevated leptin/adiponectin ratio, can directly reflect adipose tissue dysfunction and these changes may precede and predict obesity-related metabolic disorders [7], WC in conjunction with leptin/adiponectin may more accurately identify abnormal fat distribution and function in children and thus predict their risk of MetS at an earlier stage [48]. Future longitudinal studies are warranted to study these postulations.

## Conclusion

In this study, prevalence of MetS in normal weight, overweight and obese children in Beijing was estimated according to the IDF 2007 and modified ATPIII definitions. Comparisons were made between these definitions. For children ≥10 years, prevalence of MetS was 14.3% in the obese group and 3.7% in the overweight group according to the IDF definition. Factor structures of MetS with and without adipokines were investigated with exploratory factor analysis. To our knowledge, this is the first study to incorporate leptin/adiponectin ratio as an additional MetS component in a one-factor model and show that it outperforms other clinical and biochemical parameters.

This study has some limitations, which need to be considered. First, the cross-sectional design of limited our ability to comment on causal associations between the factors studied. Our ongoing follow-up study is expected to overcome this problem. Second, although a hyperinsulinemic euglycemic clamp is the gold standard method to assess insulin sensitivity, it was not practical in a large sample such as this. However, in a previous study, it has been demonstrated that fasting insulin and WC together gave results similar to insulin sensitivity measured directly by the hyperinsulinemic euglycemic clamp [20], and these two variables have been well measured in our study.

In conclusion, our results show that prevalence of MetS in overweight/obese children older than 10 years is high, regardless of the definition used. Furthermore, metabolic abnormality in children under 10 years is also worthy of concern. Factor analysis shows that central obesity is the key factor in the clustering of metabolic risk factors, while hyperleptinemia provides a connection between the different factors underlying the MetS. We suggest using leptin/adiponectin as a component of MetS. Finally, in our study a single factor has been suggested to underlie the MetS construct in Chinese children and adolescents, suggesting that there could be some pattern of common causation for the clustering of MetS components.

## Competing interests

The authors declare that they have no competing interests.

## Authors’ contributions

QXW collected the data, performed the analysis, and wrote the manuscript. JHY and LX carried out the immunoassays. HC and XYZ collected and analyzed the data. HDX and HSL contributed to reviewed/edited the manuscript. JM and ML initiated, supervised, conducted, and commented on all drafts of this paper. All authors read and approved the final manuscript.

## Pre-publication history

The pre-publication history for this paper can be accessed here:

http://www.biomedcentral.com/1471-2458/13/249/prepub

## Supplementary Material

Additional file 1: Table S1 Prevalence of other metabolic abnormalities by BMI and gender classification. Results are expressed as number with percentage (%). Significance was calculated by χ2 test or Fisher’s exact test among normal weight, over weight and obese groups in both genders. Insulin resistance was defined as HOMA-IR ≥ 3.0, equal to the 95^th^ percentile of the reference population (subjects without any traditional MetS component from BCAMS study). Hyperleptinemia was defined as leptin ≥ 95^th^ percentile and hypoadiponectinemia was defined as adiponectin ≤ 5^th^ percentile for gender of the same reference population. **Table S2**. Pearson correlation coefficients between variables associated with MetS among boys and girls. Abbreviations: MetS, metabolic syndrome; WC, waist circumference; SBP, systolic blood pressure; DBP, diastolic blood pressure; MAP, mean arterial pressure; HOMA-IR, homeostatic model assessment of insulin resistance; TG, triglycerides; HDL-C, high-density lipoprotein cholesterol; TG/HDL, TG-to-HDL ratio; leptin/adiponectin, leptin-to-adiponectin ratio. # skewed distributions logarithmically transformed for Pearson correlation. Significances are ∗P <0.05, ∗∗P < 0.01, and ∗∗∗P < 0.001. Click here for file
