# Supplementary material for: Prevalence of metabolic syndrome in a cohort of Chinese schoolchildren: comparison of two definitions and assessment of adipokines as components by factor analysis
Source: BMC Public Health. 2013 Mar 21;13:249. doi: 10.1186/1471-2458-13-249 (PMC3608951; doi:10.1186/1471-2458-13-249)
Supplement: Additional file 1: Table S1 — Prevalence of other metabolic abnormalities by BMI and gender classification. Results are expressed as number with percentage (%). Significance was calculated by χ2 test or Fisher’s exact test among normal weight, over weight and obese groups in both genders. Insulin resistance was defined as HOMA-IR ≥ 3.0, equal to the 95th percentile of the reference population (subjects without any traditional MetS component from BCAMS study). Hyperleptinemia was defined as leptin ≥ 95th percentile and hypoadiponectinemia was defined as adiponectin ≤ 5th percentile for gender of the same reference population. Table S2. Pearson correlation coefficients between variables associated with MetS among boys and girls. Abbreviations: MetS, metabolic syndrome; WC, waist circumference; SBP, systolic blood pressure; DBP, diastolic blood pressure; MAP, mean arterial pressure; HOMA-IR, homeostatic model assessment of insulin resistance; TG, triglycerides; HDL-C, high-density lipoprotein cholesterol; TG/HDL, TG-to-HDL ratio; leptin/adiponectin, leptin-to-adiponectin ratio. # skewed distributions logarithmically transformed for Pearson correlation. Significances are ∗P <0.05, ∗∗P < 0.01, and ∗∗∗P < 0.001. [file 1471-2458-13-249-S1.docx]

**Additional file 1**

Table 1- Prevalence of other metabolic abnormalities by BMI and gender classification.

|  | Boys | | | | | | | |  | | Girls | | | |
| --- | --- | --- | --- | --- | --- | --- | --- | --- | --- | --- | --- | --- | --- | --- |
|  | Normal weight | | Overweight | | Obese | | P | |  | | Normal weight | Overweight | Obese | P |
| Children ≥ 10 years | | | | | | | | | | | | | | |
| N | | 464 | | 269 | | 546 | |  | |  | 725 | 268 | 292 |  |
| Insulin resistance | | 44(9.5%) | | 82(30.5%) | | 284(52.0%) | | ＜0.001 | |  | 87(12.0%) | 95(35.4%) | 164(56.2%) | ＜0.001 |
| Hyperleptinemia | | 30(6.5%) | | 147(54.6%) | | 474(86.8%) | | ＜0.001 | |  | 67(9.2%) | 131(48.9%) | 207(70.9%) | ＜0.001 |
| Hypoadiponectinemia | | 34(7.3%) | | 29(10.8%) | | 82(15.1%) | | 0.001 | |  | 53(7.3%) | 36(13.4%) | 49(16.8%) | ＜0.001 |
| Children ＜ 10 years | | | | | | | | | | | | | | |
| N | 165 | | 50 | | 223 | |  | |  | | 187 | 50 | 134 |  |
| Insulin resistance | 6(3.6%) | | 1(2.0%) | | 45(20.2%) | | ＜0.001 | |  | | 1(0.5%) | 4(8.0%) | 30(22.4%) | ＜0.001 |
| Hyperleptinemia | 8(4.8%) | | 23(46.0%) | | 186(83.4%) | | ＜0.001 | |  | | 7(3.7%) | 6(12.0%) | 62(46.3%) | ＜0.001 |
| Hypoadiponectinemia | 0 | | 1(2.0%) | | 13(5.8%) | | 0.002 | |  | | 4(2.1%) | 1(2.0%) | 15(11.2%) | 0.001 |

Results are expressed as number with percentage (%). Significance was calculated by χ2 test or fisher’s exact test among normal weight, over weight and obese groups in both genders. Insulin resistance was defined as HOMA-IR≥3.0, equal to the 95^th^ percentile of the reference population (subjects without any traditional MS component from BCAMS study). Hyperleptinemia was defined as leptin ≥ 95^th^ percentile and hypoadiponectinemia was defined as adiponectin ≤ 5^th^ percentile for gender of the same reference population.

### Table 2- Pearson correlation coefficients between variables associated with MetS among boys and girls.

| Variables | WC | SBP | DBP | MAP | Glucose | Insulin | HOMA-IR | TG | HDL-C | TG/HDL-C | Leptin | Adiponectin | Leptin/Adiponectin |
| --- | --- | --- | --- | --- | --- | --- | --- | --- | --- | --- | --- | --- | --- |
| ***Boys*** |  |  |  |  |  |  |  |  |  |  |  |  |  |
| WC | 1 | 0.619  ∗∗∗ | 0.489  ∗∗∗ | 0.584  ∗∗∗ | 0.085  ∗∗∗ | 0.608  ∗∗∗ | 0.596  ∗∗∗ | 0.425  ∗∗∗ | -0.499  ∗∗∗ | 0.519  ∗∗∗ | 0.689  ∗∗∗ | -0.429  ∗∗∗ | 0.740  ∗∗∗ |
| SBP |  | 1 | 0.717  ∗∗∗ | 0.898  ∗∗∗ | 0.149  ∗∗∗ | 0.440  ∗∗∗ | 0.442  ∗∗∗ | 0.287  ∗∗∗ | -0.339  ∗∗∗ | 0.353  ∗∗∗ | 0.351  ∗∗∗ | -0.307  ∗∗∗ | 0.410  ∗∗∗ |
| DBP |  |  | 1 | 0.951  ∗∗∗ | 0.133  ∗∗∗ | 0.330  ∗∗∗ | 0.334  ∗∗∗ | 0.245  ∗∗∗ | -0.264  ∗∗∗ | 0.290  ∗∗∗ | 0.294  ∗∗∗ | -0.201  ∗∗∗ | 0.323  ∗∗∗ |
| MAP |  |  |  | 1 | 0.151  ∗∗∗ | 0.404  ∗∗∗ | 0.408  ∗∗∗ | 0.282  ∗∗∗ | -0.317  ∗∗∗ | 0.340  ∗∗∗ | 0.342  ∗∗∗ | -0.263  ∗∗∗ | 0.386  ∗∗∗ |
| Glucose |  |  |  |  | 1 | 0.237  ∗∗∗ | 0.346  ∗∗∗ | 0.067  ∗∗ | 0.025 | 0.055  ∗ | 0.074  ∗∗ | 0.013 | 0.058  ∗ |
| Insulin# |  |  |  |  |  | 1 | 0.993  ∗∗∗ | 0.436  ∗∗∗ | -0.376  ∗∗∗ | 0.479  ∗∗∗ | 0.572  ∗∗∗ | -0.338  ∗∗∗ | 0.608  ∗∗∗ |
| HOMA-IR# |  |  |  |  |  |  | 1 | 0.428  ∗∗∗ | -0.362  ∗∗∗ | 0.469  ∗∗∗ | 0.561  ∗∗∗ | -0.323  ∗∗∗ | 0.593  ∗∗∗ |
| TG# |  |  |  |  |  |  |  | 1 | -0.456  ∗∗∗ | 0.943  ∗∗∗ | 0.460  ∗∗∗ | -0.225  ∗∗∗ | 0.472  ∗∗∗ |
| HDL-C |  |  |  |  |  |  |  |  | 1 | -0.719  ∗∗∗ | -0.330  ∗∗∗ | 0.359  ∗∗∗ | -0.411  ∗∗∗ |
| TG/HDL-C# |  |  |  |  |  |  |  |  |  | 1 | 0.477  ∗∗∗ | -0.312  ∗∗∗ | 0.518  ∗∗∗ |
| Leptin# |  |  |  |  |  |  |  |  |  |  | 1 | -0.241  ∗∗∗ | 0.935  ∗∗∗ |
| Adiponectin# |  |  |  |  |  |  |  |  |  |  |  | 1 | -0.571  ∗∗∗ |
| Leptin/  adiponectin# |  |  |  |  |  |  |  |  |  |  |  |  | 1 |
| ***Girls*** |  |  |  |  |  |  |  |  |  |  |  |  |  |
| WC | 1 | 0.492  ∗∗∗ | 0.420  ∗∗∗ | 0.483  ∗∗∗ | 0.164  ∗∗∗ | 0.613  ∗∗∗ | 0.606  ∗∗∗ | 0.263  ∗∗∗ | -0.404  ∗∗∗ | 0.365  ∗∗∗ | 0.755  ∗∗∗ | -0.361  ∗∗∗ | 0.756  ∗∗∗ |
| SBP |  | 1 | 0.713  ∗∗∗ | 0.889  ∗∗∗ | 0.176  ∗∗∗ | 0.401  ∗∗∗ | 0.408  ∗∗∗ | 0.123  ∗∗∗ | -0.241  ∗∗∗ | 0.189  ∗∗∗ | 0.396  ∗∗∗ | -0.216  ∗∗∗ | 0.407  ∗∗∗ |
| DBP |  |  | 1 | 0.955  ∗∗∗ | 0.173  ∗∗∗ | 0.345  ∗∗∗ | 0.356  ∗∗∗ | 0.116  ∗∗∗ | -0.184  ∗∗∗ | 0.161  ∗∗∗ | 0.359  ∗∗∗ | -0.154  ∗∗∗ | 0.353  ∗∗∗ |
| MAP |  |  |  | 1 | 0.188  ∗∗∗ | 0.395  ∗∗∗ | 0.405  ∗∗∗ | 0.127  ∗∗∗ | -0.222  ∗∗∗ | 0.185  ∗∗∗ | 0.402  ∗∗∗ | -0.192  ∗∗∗ | 0.403  ∗∗∗ |
| Glucose |  |  |  |  | 1 | 0.248  ∗∗∗ | 0.368  ∗∗∗ | 0.131  ∗∗∗ | -0.082  ∗∗ | 0.135  ∗∗∗ | 0.141  ∗∗∗ | -0.065  ∗∗ | 0.140  ∗∗∗ |
| Insulin# |  |  |  |  |  | 1 | 0.991  ∗∗∗ | 0.318  ∗∗∗ | -0.282  ∗∗∗ | 0.360  ∗∗∗ | 0.624  ∗∗∗ | -0.290  ∗∗∗ | 0.621  ∗∗∗ |
| HOMA-IR# |  |  |  |  |  |  | 1 | 0.319  ∗∗∗ | -0.280  ∗∗∗ | 0.360  ∗∗∗ | 0.617  ∗∗∗ | -0.284  ∗∗∗ | 0.613  ∗∗∗ |
| TG# |  |  |  |  |  |  |  | 1 | -0.373  ∗∗∗ | 0.932  ∗∗∗ | 0.330  ∗∗∗ | -0.179  ∗∗∗ | 0.339  ∗∗∗ |
| HDL-C |  |  |  |  |  |  |  |  | 1 | -0.679  ∗∗∗ | -0.330  ∗∗∗ | 0.308  ∗∗∗ | -0.390  ∗∗∗ |
| TG/HDL-C# |  |  |  |  |  |  |  |  |  | 1 | 0.385  ∗∗∗ | -0.260  ∗∗∗ | 0.416  ∗∗∗ |
| Leptin# |  |  |  |  |  |  |  |  |  |  | 1 | -0.285  ∗∗∗ | 0.925  ∗∗∗ |
| Adiponectin# |  |  |  |  |  |  |  |  |  |  |  | 1 | -0.628  ∗∗∗ |
| Leptin/  adiponectin# |  |  |  |  |  |  |  |  |  |  |  |  | 1 |

Abbreviations: MS, metabolic syndrome; WC, waist circumference; SBP, systolic blood pressure; DBP, diastolic blood pressure; MAP, mean arterial pressure; HOMA-IR, homeostatic model assessment of insulin resistance; TG, triglycerides; HDL-C, high-density lipoprotein cholesterol; TG/HDL, TG-to-HDL ratio; leptin/adiponectin, leptin-to-adiponectin ratio. # skewed distributions logarithmically transformed for Pearson correlation. Significances are ∗P <0.05, ∗∗P < 0.01, and ∗∗∗P < 0.001.
